# Four Operations for Lithotomy on Same Patient

**Published:** 1866-07

**Authors:** 


FOUR OPERATIONS FOR LITHOTOMY ON SAME PATIENT.

Dr. J. C. Hughes, of Iowa, reported a case of vesical calculi, in which he had four times performed the bilateral section in the same patient. When the second operation became necessary, lithotrity was attempted, but failed. At the time of the third operation the patient was much emaciated and very feeble, yet he recovered nicely. In less than another year the fourth operation was demanded, when twelve calculi were removed; drawings of these accompanying the papers. Dr. Hughes spoke highly of the bilateral as compared with the lateral operation, having performed it twenty-one times with the loss of but a single patient.

The paper was referred to Publication Committee.

Dr. Hughes also spoke of a new method of operating upon limbs which were both curved and shortened. He said Barton's mode was to saw out the V-shaped portion of the bone on its convexity; but as Dr. Hughes wished both to straighten and lengthen the limb, he cut down on the convex side, and passing a chain-saw around the bone, made a partial section on the side of its concavity, and then used such manual force as was necessary to straighten it—thus leaving a chasm to be filled instead of losing an additional inch.

It was moved by Dr. H. R. Storer, of Mass., that Dr. Hughes be requested to write a description of the operation for insertion in the Transactions of this Association.

On motion, the section adjourned to meet at 3 o'clock P.M., to-morrow, May 3d, in the same place.

*Third Session, May 3.*

The Section met at the appointed hour. Dr. Post in the chair.

Dr. Lewis A. Sayre, of New York, moved that Dr. Post be requested to furnish for the Transactions of the Association a description of his grooved needle, previously exhibited. Dr. Sayre thought the thanks of the profession were due to Dr.

Post for this unpretending but eminently useful instrument. Carried.

Dr. Sayre then read a paper on Luxation of the Femur into the Ischiatic Notch, of Nine Months' Standing, reduced by manipulation.

Referred to Publication Committee.

Dr. Dewit C. Enos, of Brooklyn, read a paper on the Intraoral Method of operating for Removal of Lower Jaw.

Referred to Publication Committee.

Dr. George M. McGill, U.S.A., presented a paper advocating the adoption of a periosteum flap in all amputations in the continuity—including a report of five cases. The Dr. exhibited casts of two of the stumps after these operations, which were beautifully oval.

Paper referred to Publication Committee.

Dr. Enos thought the suggestion of Dr. McGill a good one, but in one case in which he had made a periosteal flap in his own practice there had been such a reproduction of bone as to necessitate a second operation.

Dr. Louis Elsberg, of New York, read a paper on the Means of Diagnosis at present available in Diseases of the Larynx, with full directions for the use of Laryngoscope.

On motion, referred to Publication Committee.

Dr. B. J. Raphael, of New York, stated that Dr. Montrose A. Polk, of ——, made a partial report in 1860, on ——, and moved that the committee be continued. Carried. On motion of Dr. Raphael, Dr. E. Krackowizer, of New York, and Dr. J. S. Cohen, of Philadelphia, were appointed a Committee to report on the subject of Local Anæsthesia. Dr. Post made some remarks on the subject of partial anæsthesia and the coöperation of the intelligent patient with the practitioner.

On motion of Dr. B. J. Raphael, Dr. Henry D. Noyes, of New York, was appointed a committee to report on the influence upon vision of the abnormal conditions of the muscular apparatus of the eye.

On motion of Dr. E. Krackowiser, of New York, Dr. Raphael was appointed a committee to report on the comparative merits of the different operations for the extraction of vesicular calculi.

Whereupon the Section adjourned *sine die.*

